# Transplant Tolerance: New Insights and Strategies for Long-Term Allograft Acceptance

**DOI:** 10.1155/2013/210506

**Published:** 2013-05-12

**Authors:** Paulina Ruiz, Paula Maldonado, Yessia Hidalgo, Alejandra Gleisner, Daniela Sauma, Cinthia Silva, Juan Jose Saez, Sarah Nuñez, Mario Rosemblatt, Maria Rosa Bono

**Affiliations:** ^1^Departamento de Biologia, Facultad de Ciencias, Universidad de Chile, 7800024 Santiago, Chile; ^2^Programa de Ciencias Biomedicas, Facultad de Medicina, Universidad de Chile, 8380453 Santiago, Chile; ^3^Fundacion Ciencia y Vida, 7780272 Santiago, Chile; ^4^Facultad de Ciencias Biologicas, Universidad Andres Bello, 8370146 Santiago, Chile

## Abstract

One of the greatest advances in medicine during the past century is the introduction of organ transplantation. This therapeutic strategy designed to treat organ failure and organ dysfunction allows to prolong the survival of many patients that are faced with no other treatment option. Today, organ transplantation between genetically dissimilar individuals (allogeneic grafting) is a procedure widely used as a therapeutic alternative in cases of organ failure, hematological disease treatment, and some malignancies. Despite the potential of organ transplantation, the administration of immunosuppressive drugs required for allograft acceptance induces severe immunosuppression in transplanted patients, which leads to serious side effects such as infection with opportunistic pathogens and the occurrence of neoplasias, in addition to the known intrinsic toxicity of these drugs. To solve this setback in allotransplantation, researchers have focused on manipulating the immune response in order to create a state of tolerance rather than unspecific immunosuppression. Here, we describe the different treatments and some of the novel immunotherapeutic strategies undertaken to induce transplantation tolerance.

## 1. History of Organ Transplantation

Earl C. Padgett first described the phenomenon of allograft rejection in 1932. He used nonrelated skin allografts to cover severely burned patients and reported that none of the skin allografts survived permanently. However, he observed that skin grafts from relatives seemed to survive longer than those from unrelated donors [1]. In 1943, Gibson and Medawar developed the first scientific explanation of the phenomenon of allorejection. They observed that patients who received autografts (tissue from the same individual transplanted to a different part of the body) accepted the tissue with no complications unlike patients that had received a sibling's skin allograft (tissue from a different individual belonging to the same species) who eventually rejected the allograft. In addition, they observed that a second skin transplant with skin from the same donor resulted in more rapid rejection compared with the first skin transplantation. The observation of the accelerated rejection of the second graft from the same donor was convincing evidence that supported the involvement of an immunological process during allograft rejection [2, 3]. 

In 1948, Medawar and colleagues excluded an important role of antibodies in allograft rejection [4, 5] and designed an experiment to assess whether cellular components of the immune system are responsible for transplant rejection. They injected cells from the allograft-draining lymph node from transplanted mice into mice recently transplanted with skin from the same donor. They observed that mice rejected the allograft as similar to mice transplanted for a second time, indicating that cellular components of the immune system are responsible for the generation of the immune response against the allograft [3, 6]. 

Advances achieved in surgical techniques in parallel with improvements in knowledge of the immune mechanisms mediating allograft rejection allowed the first kidney transplant in 1963 [7–10]. Joseph E. Murray and his colleagues at Peter Bent Brigham Hospital in Boston performed the first successful kidney transplant from one twin to another [11]. It was a great advance in medicine, demonstrating that it was possible to perform successful organ transplants in humans, but it was still necessary to solve the problem of rejection between unrelated donors [12]. 

Since then, different pharmacological treatments have been developed in order to induce an immunosuppressive state that allows the acceptance of an allograft transplant between unrelated donors [1, 13–16]. The first successful cadaveric unrelated kidney transplant was performed in 1962 by Joseph Murray and his group [17]. Murray used azathioprine, an immunosuppressive drug previously tested in dogs [18], which allowed the transplant recipient to survive one year after receiving the kidney transplant [17, 19]. 

The immunosuppressive effects of cyclosporine A (CsA) were discovered in Switzerland in 1972. Some trials to compare CsA versus azathioprine and steroids were developed and the promising results led to clinical approval for the use of CsA in human transplants in 1980 [20, 21]. The introduction of CsA contributed substantially towards the improvement of allograft and patient survival [22].

The massive development of immunosuppressive drugs opened the door to organ transplantation, extending to other organs such as the liver, lungs, and heart. In parallel with the increased number of organ transplants, several investigators are currently working on developing new immunosuppressive drug protocols that will further improve the outcome and reduce tissue toxicity in transplanted patients [23–26]. However, despite these efforts, currently all immunosuppressive drugs have serious side effects including nephrotoxicity, development of malignancies, and susceptibility to infections by opportunistic pathogens. For this reason, immunologists face a new challenge in developing strategies to reduce or eliminate the use of immunosuppressive drugs in organ transplants. These efforts are being focused on reeducating the immune system or inducing allograft-specific tolerance mechanisms. 

## 2. Immune Tolerance

One of the hallmarks of the adaptive immune system is its ability to recognize a vast number of different antigens. This ability is a consequence of the large lymphocyte repertoire, in which each cell has a different antigen receptor generated by the process of somatic recombination. This process is able to produce an estimate of 10^15^ different lymphocyte clones, each with a different antigen receptor that can hypothetically recognize any naturally occurring structure [27]. Since somatic recombination is a random process, it generates T cell clones that can recognize self-structures or self-peptides (auto-antigens). The mechanism used by the immune system in order to avoid a possible harmful immune response against an individual's own cells and tissues is known as immune tolerance and can be classified into central and peripheral tolerance. 

### 2.1. Central Tolerance

Central tolerance occurs in the thymus and allows the deletion of a major percentage of auto-reactive T cells. The thymus is the major site of maturation of T cells and can be anatomically and functionally separated into two zones: the thymic cortex and medulla. The cortex is the region where the process of positive selection occurs and contains densely packed immature thymocytes. The medulla contains loosely packed mature lymphocytes and is the site where the process of negative selection takes place [28]. 

#### 2.1.1. Positive Selection

After originating in the bone marrow, the early precursors of T cells enter the thymus and migrate into the cortex where most of the subsequent maturation events take place. These T cell precursors do not express the T cell receptor (TCR), CD3, *ζ* chains, CD4, or CD8 coreceptors and therefore are called CD4^−^CD8^−^ double negative (DN) thymocytes. Within the cortex, DN cells undergo TCR rearrangement and become CD4^+^CD8^+^ double positive (DP) cells, which express the TCR *α* and *β* chains as well as both CD4 and CD8 coreceptors. 

#### 2.1.2. Negative Selection

Double positive cells are programmed to undergo apoptosis by default unless they receive a “rescue signal” which is provided by cortical thymic epithelial cells (cTEC) that express self-peptide/major histocompatibility complex (MHC). Only thymocytes recognizing self-peptide/MHC complex with low avidity will receive the rescue signals and will continue with the maturation process. The DP clones that are rescued will continue with the process of maturation and will become single positive (SP) cells that express either the CD4 or CD8 coreceptor [29–31]. 

The acquisition of adequate chemokine receptors allows SP cells to exit the thymic cortex and to enter the medulla. It is in the medulla where they will continue with the negative selection process, which is crucial to central tolerance [29, 32, 33].

One of the questions regarding negative selection is how autoreactive clones that recognize self-peptides that are not normally found in the thymus are controlled. Recent evidence has demonstrated that the AIRE transcription factor is involved in the promiscuous gene expression in mTEC cells that allows an increase in the repertory of auto-antigens presented by antigen presenting cells (APCs) during negative selection [34–38].

As a consequence of positive and negative selection, T cells that leave the thymus and populate peripheral lymphoid tissues are self-MHC restricted and tolerant to many auto-antigens.

### 2.2. Peripheral Tolerance

Although central tolerance mechanisms are efficient in deleting the auto-reactive T cell clones that recognize self-antigen/MHC complex with high affinity, some autoreactive T cells are able to bypass this control and exit the thymus [39–41]. In the periphery, these auto-reactive clones are able to induce autoimmune responses, generally in response to an inflammatory environment such as one triggered during infection [42, 43]. Therefore, there is a constant threat of potential autoimmune responses due to the escape of auto-reactive T cells clones to the periphery. These potentially harmful auto-reactive cells must be effectively controlled by peripheral tolerance mechanisms.

Peripheral tolerance mechanisms involve the *deletion* of activated effector T cells, *anergy* induction, *clonal exhaustion,* and active *regulation* of effectors T cells [44]. Regulatory T cells (Tregs) mediate active regulation of the immune response preventing autoimmune and inflammatory diseases and restraining responses to infections of viral, bacterial, or parasitic origin. Moreover Tregs can restrain immune responses directed towards tumors or transplanted tissue [42–46].

Two different types of Tregs have been described; natural CD4^+^CD25^+^Foxp3^+^ regulatory T cells (nTregs), which are generated in the thymus and regulate immune responses in the periphery, and inducible CD4^+^CD25^+^Foxp3^+^ regulatory T cells (iTregs) which develop in the periphery from naïve CD4^+^ T cells after exposure to antigens in a specific cytokine microenvironment, tolerogenic APCs, or immunosuppressive drugs [44].

Dendritic cells play an important role in establishing peripheral tolerance. These cells are found in mucosal and parenchymal tissues where they function as sentinels in search for pathogens and tissue injury. During infection and tissue damage, immature DCs (iDCs) are activated through different pathogen-associated molecular pattern (PAMP) receptors, which trigger the maturation of DCs. These DCs migrate to the draining lymph nodes where they acquire the capacity to activate naïve T cells [39]. Under steady-state conditions, iDCs constitutively take up and process cellular debris produced as a consequence of normal cell turnover of the tissues. Internalization of self-antigens present in apoptotic cells by peripheral iDCs induces tolerance mechanisms such as the expansion of iTregs that control effector responses and protect cells and tissues from damage during pathogenic autoimmunity [47]. 

## 3. Mechanisms of Allograft Rejection

### 3.1. Clinical Rejection

Despite the advances in transplantation tolerance, the mechanisms that mediate allograft rejection have not yet been fully described. Clinical rejection may occur at any time following transplantation and therefore is classified according to the time in which it occurs after the transplant. 

*Hyperacute rejection* may occur within a few minutes to hours after transplantation. It is due to preformed alloantibodies by the recipient, mainly against MHC antigens, which become deposited in the allograft and induce complement activation and recruitment of inflammatory cells that trigger platelet aggregation, with consequent capillary obstruction and tissue necrosis. This type of rejection is not very common nowadays because it is easily prevented by blood typing and crossmatching prior to transplantation. 

*Acute rejection* occurs days to months after the transplant. It consists of a tissue injury process mediated by alloantibodies and alloreactive T cells, mainly in response to MHC antigens. Acute cellular rejection is due to alloreactive cytotoxic CD8^+^ T cells that recognize the alloantigens present in the transplanted tissue and carry out its destruction. The lesion occurs mostly in the endothelial cells, which in response to the injury develop a microvascular endothelialitis and arteritis. Antibody-mediated rejection, on the other hand, is characterized by alloantibodies that induce complement activation, neutrophil recruitment, and the consequent inflammation and coagulation activation that results in thrombotic ischemia of the transplanted tissue. This type of rejection was a critical obstacle to overcome in the early steps of organ transplantation; however, today it is well managed by the employment of immunosuppressive drugs.

*Chronic rejection* is today the main cause of allograft failure. It occurs months or years following transplantation. Organ failure occurs due to chronic inflammation that triggers the proliferation of intimal smooth muscle cells and results in vascular occlusion and ischemic damage. The pathogenesis involves the chronic secretion of cytokines by activated T lymphocytes and the production of alloantibodies that are able to activate the complement system through the classical pathway, thus generating chronic damage [48]. Despite the advances in immunosuppressive therapy, this type of rejection remains unresolved [49, 50] and it is necessary to develop new strategies to improve organ acceptance.

As mentioned above, alloantibodies have an important role in the different types of rejection mechanisms. These antibodies can be directed against HLA (major antigens) or non-HLA molecules (minor antigens). Therefore it is important to detect their presence in order to prevent possible events of organ rejection. 

### 3.2. Immune Mechanisms of Rejection

#### 3.2.1. Ischemic-Reperfusion Injury

When the allograft is recovered from the donor, the organ has to undergo a procedure that necessarily involves the induction of stress. The different sources of stress during the medical procedure, namely, anesthesia, damage by physical factors (temperature and mechanical stress), and ischemia trigger an inflammatory state called “ischemic-reperfusion injury” (IRI). IRI induced by organ manipulation induces the expression of danger-associated molecular patterns (DAMPs), such as heat-shock proteins or HGMB1 that are recognized by pattern recognition receptors (PRRs) localized on epithelial cells and cells of the immune system such as neutrophils, macrophages, and DCs [51, 52].

The recognition of DAMPs by PRRs results in the activation of signaling pathways that activate the inflammasome, that is, synthesis of transcription factors and micro-RNAs, that results in an inflammatory response. The secretion of inflammatory cytokines such as a interleukin (IL)-1 and IL-6, as well as chemokines, and also the complement cascade activation [53] contribute to the generation of a microenvironment required to activate DCs. Activated DCs carrying the alloantigens from the transplanted organ then migrate to the lymph nodes and induce the activation of alloantigen-specific T cells, thus mounting a specific immune response against the allograft [54]. 

#### 3.2.2. Allorecognition and T Cell Activation Mechanisms

Today, the cellular events involved in organ rejection are better understood and three key mechanisms have been described that explain the activation of T cells by alloantigens, resulting in allograft rejection. The first mechanism of alloantigen recognition is called *direct presentation*. Donor APCs, mainly DCs present in the allograft, mediate this type of presentation. These donor DCs migrate to the draining lymph nodes where they present alloantigens (in the context of donor MHC molecules) to alloreactive recipient T cells [54]. This type of allopresentation is responsible for the activation of the immune system against the donor allograft in acute rejection. However, this allorecognition mechanism is not permanent since donor DCs are cleared out over time, due to natural cell death.

*Indirect presentation* is mediated by recipient DCs that process and present different alloantigens from the graft to alloreactive recipient T cells. In contrast with direct presentation, the alloantigens presented by DCs in indirect presentation are processed as exogenous antigens and are therefore presented by APCs in a self-MHC context. This type of alloantigen presentation is responsible for the aforementioned chronic rejection and therefore is the main cause of organ loss, which currently cannot be addressed through prevention or treatment. 

**Figure 1:**
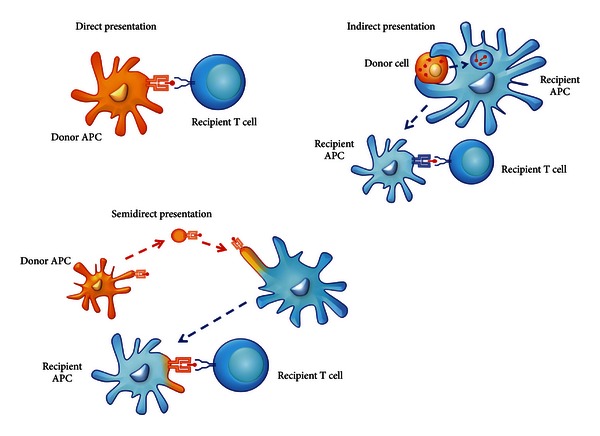
Mechanisms of alloantigen recognition. In direct presentation, donor APCs are able to present alloantigens to alloreactive T cells from the recipient. In indirect presentation, alloantigens are taken up from donor cells by recipient DCs that process and present alloantigens to alloreactive T cells. In semidirect presentation, intact MHC molecules are transferred to recipient DCs that directly present alloantigens through donor-MHC or process and present alloantigens as described for indirect presentation.

The third mechanism involved in allograft recognition is called *semidirect presentation* where donor membrane fragments which carry MHC class I molecules among others are transferred to recipient APCs (Figure 1) [55, 56]. Semidirect presentation is likely to involve cell-to-cell interaction, or release and uptake of small MHC-containing vesicles [57]. 

## 4. New Strategies to Induce Long-Term Acceptance to Organ Transplantation

The immune system protects the host from a broad range of pathogens by generating a response mediated by T cells, B cells, and innate immune cells. After the clearance of the pathogen, immune regulation avoids misguided or excessive immune reactions that could damage self-tissues, maintaining or restoring a homeostatic environment. The state of unresponsiveness of the immune system to antigens is known as immune tolerance, and this involves tolerance to self-antigens, which is established and maintained to avoid host damage.

In transplanted patients, prevention of graft rejection is achieved by long-term use of immunosuppressive drugs, which have an effect over the entire immune system, rather than a specific effect over alloreactive T cells. The development of new drugs and protocols of drug combinations is in continuous progress, but drug toxicity, chronic rejection, and immune deficiencies associated with these treatments remain unresolved. Current research is focused on promoting allograft-specific immune tolerance as a means to reduce the dose and number of immunosuppressive drugs administered, thereby allowing the host to react to potential pathogens and malignancies. 

The two major approaches to induce transplant tolerance involve, first, the induction of a state of mixed chimerism through the transfer of donor hematopoietic stem cells (HSC) to the recipient, thereby inducing central tolerance to alloantigens and, second, the delivery of alloantigens to the recipient in a “tolerogenic fashion” in order to activate peripheral tolerance mechanisms to the allograft. In the following sections, we will discuss the current research that is being carried out concerning new strategies to induce long-term acceptance of allografts. 

### 4.1. Mixed Chimerism as a Strategy to Induce Allograft Tolerance

Mixed chimerism is defined as the coexistence of donor and recipient hematopoietic cells in an individual after allogeneic bone marrow transplantation (BMT) [58, 59]. To be considered mixed chimerism, donor cells in the blood must represent more than 1% of the total cells as measured by flow cytometry [58, 60]. To induce a state of mixed chimerism, it is necessary to perform a conditioning treatment in order to allow donor HSC bone marrow acceptance. The establishment of mixed chimerism allows the redefinition of immunological “self” previously learned in the thymus. The allogeneic BMT generates a new source of T cells and DCs that induces a relearning of the “new self” state, depleting the possible T cell clones that recognize both allo- and autoantigens [58]. 

Currently used mixed chimerism protocols induce robust donor-specific tolerance and allow long-term acceptance of fully mismatched skin grafts in murine models [61]. Tolerance maintenance is mediated by intrathymic clonal deletion of alloreactive cells [62–64], mimicking the natural mechanisms to produce self-tolerance. Deletion of host alloreactive T cells depends on the continuous presence of donor DCs in the thymus [62, 63, 65], while donor alloreactive T cells are eliminated intrathymically by clonal deletion. Thus, the new T cell repertoire in chimeras is tolerant to both recipient and donor cells. 

Evidence of tolerance induction due to mixed chimerism has been reported in kidney transplant patients. Patients who had received a conventional BMT (usually to treat a hematological malignancy) that later developed organ failure accepted an organ transplant from the same donor with the use of myeloablative conditioning (elimination of recipient HSC). Such patients are able to accept the transplanted organ even across MHC barriers [66–70]. However, myeloablative conditioning is not ethically accepted due to the high risk involved in this type of conditioning.

Nonmyeloablative conditioning has emerged as an alternative to produce tolerance through mixed chimerism. Nonmyeloablative conditioning consists of the administration of sufficient immunosuppression (e.g., antithymocyte globulin, costimulation blockage, and immunosuppressive drugs) to allow the engraftment of fully mismatched BMT, but at the same time, minimal enough to avoid toxic secondary effects. Although some physical and pharmacological strategies such as total body irradiation, thymic irradiation, or the use of depleting antibodies are able to induce mixed chimerism; however, it is still necessary to generate conditioning protocols that minimize systemic immunosuppression [58, 71–74]. 

New approaches have been developed in human and nonhuman primate models in order to induce mixed chimerism in nonmyeloablative conditioning protocols. Using a simultaneous bone marrow and kidney transplantation and a preconditioning protocol consisting in the costimulatory blockade with anti-CD154 antibody, Kawai and coworkers achieved the establishment of mixed chimerism and prolonged renal allograft survival in nonhuman primates [75]. Additionally, using a high-dose BMT and costimulatory blockade, it has been demonstrated the achievement of bone marrow engraftment without cytoreduction in mice [76]. In human, Kawai and coworkers have reported tolerance induction across HLA-mismatched barriers with a periconditioning treatment using pharmacological immunosuppression and thymic irradiation. This protocol allowed the removal of long-term immunosuppressive therapy achieving full acceptance of the transplanted organ up to five years after transplant [77]. However, one of the main obstacles in the induction of mixed chimerism using the aforementioned protocol is the presence of the memory T cells that can cross-react with alloantigens [78]. Recently the group of Yamada demonstrated the induction of “delayed tolerance” by performing first, a kidney transplant and second, bone morrow transplantation in addition with CD8^+^ memory T cell depletion therapy [79]. 

The use of cellular therapy in nonmyeloablative conditioning protocols could be a valuable strategy to induce mixed chimerism. The principal candidates are immature DCs, regulatory macrophages, apoptotic cells, regulatory T cells, and mesenchymal stromal/stem cells due to their capacity to induce tolerance in antigen-specific fashion, therefore minimizing the possible side effects of non-antigen-specific experimental protocols to achieve mixed chimerism. 

### 4.2. Dendritic Cells and Regulatory Macrophages

Dendritic cells constitute a heterogeneous population of professional, bone-marrow-derived APCs that have the potential to induce both tolerance and immunity [80, 81]. This potential is directly related to DC maturation status, where T cell tolerance is induced by immature DCs that express low surface levels of MHC class II and costimulatory molecules, whereas T cell immunity is generated by mature DCs that express higher levels of these antigen presenting and costimulatory molecules [82].

Dendritic cells have been well characterized in the context of organ transplantation, where it has been hypothesized that tolerogenic DCs are involved in graft acceptance while immunogenic DCs are key to graft rejection [83]. It has been described that tolerogenic DCs have the capacity to induce or expand Tregs [81, 83–87]. Tolerogenic DCs have been characterized by low levels of expression of CD86, CD40, PD-L2, and high levels of expression of PD-L1 [83, 88–90] and CD80 [91, 92]. 

A wide variety of strategies and pharmacological agents have been used to generate tolerogenic DCs *in vitro*. Such approaches include the use of cytokines and growth factors (IL-10, TGF-*β*, GM-CSF) during their differentiation, genetic interference with NF-*κ*B signaling and costimulatory molecules, and exposure to immunosuppressive agents such as CsA, vitamin D3, rapamycin, aspirin, mycophenolate mofetil, sanglifehrin A, deoxyspergualin, and corticosteroids [80, 81, 93–95]. 

IL-10-treated DCs or DCs genetically modified to overexpress IL-10 induce antigen-specific T cell anergy [96], while very low doses of GM-CSF lead to the development of immature DCs that induce alloantigen-specific T cell unresponsiveness *in vitro* and *in vivo* [97]. It has been described that the culture supernatant obtained from the GM-CSF producing-J558L cell line can be used in order to differentiate and expand immature DCs from bone marrow precursors. Some reports and our unpublished results [98, 99] demonstrated that this supernatant contains similar amounts of GM-CSF and IL-10 and that DCs generated with this supernatant have an immature/tolerogenic phenotype, since they are resistant to lipopolysaccharide (LPS) activation. This demonstrates the importance of immunomodulatory cytokines such as IL-10 in the maturation state of DCs. 

On the other hand, the immunosuppressive drugs CsA, tacrolimus, and LF15-0195 inhibit DC maturation by blocking NF-*κ*B signaling [93]. Both vitamin D3 and dexamethasone affect DC differentiation by downregulating their capacity to secrete IL-12p70, which leads to the induction of IL-10-secreting Tregs. In addition, vitamin D3-induced upregulation of PD-L1 in DCs provides inhibitory signals that regulate both central and peripheral tolerance [100] and, importantly, blockade of PD-L1 abolishes the tolerogenic capacity of vitamin D3-generated DCs [93]. Other immunosuppressive drugs such as rapamycin confer DC resistance to maturation in response to a proinflammatory stimulus [101] and promote organ transplant tolerance by inducing the *in vitro* and *in vivo* generation of Tregs [87, 93]. 

Immature DCs are also used in the generation of mixed chimerism as a strategy to induce transplant tolerance. In mouse models, mixed chimerism and transplant tolerance to a secondary skin allograft in an alloantigen-specific fashion were achieved using sequential doses of irradiated immature DC in bone marrow transplant protocols [102], demonstrating a potential use of DC in future treatments. 

In the context of transplants, macrophages have been usually associated with graft rejection and resistance to tolerance induction. It has been demonstrated that these cells are major constituents of inflammatory infiltrates and are a prominent cell type in rejecting allografts [103]. Macrophages are also able to infiltrate heart allografts and contribute to transplant vasculopathy in an animal model of chronic allograft rejection [104]. Moreover, it has been demonstrated that some kidney transplant patients experience episodes of acute rejection even in the presence of T cell depletion therapies and this type of rejection was associated with intense monocytic infiltrations [105]. All these pieces of evidence presented so far support a key role for macrophages in graft damage and rejection [106].

However, in addition to classically activated (M1-polarized) macrophages that promote Th1-type T cell responses and alternatively activated (M2-polarized) macrophages that produce IL-10 and favor Th2-polarized T cell responses, novel macrophage populations with T cell-suppressive properties called “regulatory macrophages” have been described in the literature. The group of Mosser demonstrated that stimulating macrophages in the presence of high-density immune complexes and a TLR ligand resulted in IL-10 producing macrophages [107]. On the other hand, Brem-Exner and coworkers have observed that when macrophages are driven to an activated state by the addition of IFN-*γ*, these macrophages prevent autoimmune colitis by inducing and expanding Foxp3^+^ Tregs [106, 108].

Since the discovery of these subsets of “regulatory macrophages,” much attention has been paid towards the potential use of these populations in the induction of tolerance in transplants. Evidence directly involving macrophages in the acceptance of transplants was obtained from mice injected with CSF-1 before the transplant. In this study, CSF-1 induced the expansion of the host macrophage pool, reduced donor T cell expansion, and improved GVHD morbidity and mortality after allogeneic hematopoietic cell transplantation [109]. Moreover, *in vitro* generated murine regulatory macrophages have demonstrated to completely suppress polyclonal T cell proliferation through an inducible-nitric-oxide-synthase- (iNOS-) dependent mechanism and the administration of these cells before transplantation significantly prolonged allograft survival in fully immunocompetent recipients in a heterotopic heart transplant model [110]. Recently, human regulatory macrophages were isolated from peripheral blood and characterized by their morphology, cell-surface phenotype, and their capability to inhibit T cell proliferation *in vitro* [54]. These cells have been used in kidney transplantation in human, and their utilization allowed to decrease the level of immunosuppressive drugs to induce operational tolerance to the allograft [111]. All these studies suggest that regulatory macrophages may be used as a potential immune-conditioning therapy for use in solid-organ transplantation in the future.

### 4.3. Exosomes and Phagosomes as Tools for Alloantigen Delivery

The delivery of alloantigens in a non-immunogenic context constitutes an alternative strategy to reduce the immune response following transplantation since it has been observed that donor-specific allograft tolerance can be induced in rodents by presentation of donor MHC antigens before transplantation [112]. Recent approaches include the use of exosomes and phagosomes as tools for delivering such alloantigens [86, 113–117].

Exosomes are cell-derived membrane nanovesicles of relatively uniform shape and size (50–100 nm) that can be easily purified from fluids (serum, urine, bronchoalveolar lavage, etc.) by ultracentrifugation [117–120]. Exosomes are formed by reverse budding of the limiting membrane of late endosomes/multivesicular bodies (MVB) fused to the plasma membrane. Exosomes are produced by multiple cell types such as enterocytes, mast cells, DCs, T and B lymphocytes, macrophages, tumor cells, and platelets [121–123]. 

It has been demonstrated that incubation of DCs with exosomes that carry MHC class II results in an efficient stimulation of T cells even when the DCs are MHC class II-deficient [114, 116]. On the other hand, exosomes from thymocytes have the capacity to induce Tregs that suppress the proliferation of effector T cells *in vitro* and *in vivo* [117]. 

The use of exosomes in a cardiac allograft transplant model in rats has produced promising results. Treatment with exosomes induced a significant prolongation of allograft survival, and in some recipients long-term graft survival was seen after transplantation [112]. Other reports demonstrate that exosomes derived from mature DCs can trigger effector T cell responses leading to rapid skin graft rejection, while exosomes obtained from immature DCs significantly prolong heart allograft survival [113, 115]. Moreover, a combination of donor exosomes with suboptimal doses of the immunosuppressive drug LF15-0195 induced long-lasting survival of cardiac allografts [113]. These reports demonstrate that exosomes constitute a potentially powerful tool of alloantigen delivery in order to induce immune tolerance in transplantation.

Recently, a protocol of alloantigen administration based on phagosomes has been developed. Phagocytosis of PLGA (polylactic-co-glycolic acid) nanoparticles by immature DCs allows these particles to become sequestered in the phagosome. These PLGA-containing phagosomes display a biochemical composition similar to the plasma membrane of the original phagocytic cell [86, 124]. Therefore, the disruption of PLGA-loaded immature DCs produces PLGA-phagosomes that carry alloantigens and other surface molecules expressed by immature DCs [86]. When these phagosomes are fed to immature DCs from a different strain, almost all DCs were able to capture the phagosomes while remaining immature. DCs expressed low expression levels of MHC class II and CD86 maturation markers, secreted low levels of the activating cytokines IL-2 and IL-12, and showed increased IL-10 secretion [86]. Moreover, *in vivo* studies in mice demonstrated that, when administered intravenously, PLGA-phagosomes were phagocytosed only by spleen DCs and this process did not induce DCs maturation. Additionally, when PLGA-phagosomes were used to treat mice prior to alloimmunization, there was a significant reduction in alloantibody secretion and cellular responses. This effect is specific, since third party allogeneic PLGA-phagosomes did not decrease the alloimmune response (our unpublished results). The decreased humoral and cellular immune responses observed in mice treated with phagosome-based alloantigen delivery prior to alloimmunization constitute important observations that should stimulate the use of allogeneic PLGA-phagosomes as a suitable tool for alloantigen administration in a tolerogenic context. 

### 4.4. Apoptotic Cells

The finding that apoptotic cells exert potent anti-inflammatory and immunoregulatory effects on APCs of the immune system [125] has paved the way for the development of novel apoptotic cell-based therapies that have been used successfully in delaying transplant rejection and treating T cell-mediated autoimmune disorders in murine experimental models.

Cell death is an integral cellular process that occurs by two major events: apoptosis and necrosis. Apoptosis, or programmed cell death, is an energy-dependent process that involves typical cellular morphological changes including cell shrinkage, nuclear condensation, DNA fragmentation, and membrane blebbing. Scattered cells in a tissue undergo apoptosis triggered by stimuli in both physiological and pathological conditions. In contrast, necrosis, associated with pathological tissue injury, is characterized by rapid, disorganized swelling and subsequent release of intracellular components into the local environment [126]. These different pathways leading to cell death may give rise to distinct immunological responses [126, 127]. Generally, apoptotic cells are removed through phagocytosis by resident macrophages and DCs, restraining inflammatory or immune reactions [128], and can actively promote anti-inflammatory and tolerogenic signals [47]. In contrast, debris from cells that die prematurely by necrosis is able to activate proinflammatory and immunostimulatory responses [129]. 

The molecular mechanisms that guide the recognition of apoptotic cells by phagocytes are complex and have not been entirely elucidated. Apoptotic cells display a series of apoptotic cell-associated molecular patterns (ACAMPs) that serve as “eat me” signals that are recognized by PRRs expressed on the surface of the phagocytes, including DCs [130, 131]. Under steady-state conditions, peripheral DCs take up self-antigens carried by apoptotic cells and induce a state of tolerance that protects cells and tissues from potential damage by pathogenic autoimmune reactions as well as immune responses induced by viral and bacterial infections [47]. A broad variety of factors are likely to determine whether a DC becomes tolerogenic or immunogenic after the uptake of apoptotic cells. For example, it is known that early stage apoptotic cells are more likely to induce tolerance than late stage apoptotic cells [132–134]. Molecules displayed on the surface of apoptotic cells [135], the number of apoptotic cells [136], receptors and secreted cytokines [126, 137], the presence or absence of danger signals [138], and interactions with other cells [47] can all contribute to determine different types of immune responses. Additionally, DC maturation status can play a role in the induction of tolerogenicity or immunogenicity. Immunogenic responses are generally associated with mature DCs, which display high numbers of MHC class II and costimulatory molecules. However, it has been difficult to establish a correlation between the maturity state of a DC and its tolerance-inducing function. Early evidence has indicated that tolerance in the periphery is controlled by immature DCs [139]. However, it is becoming clear that semimature and mature DCs can also induce antigen-specific tolerance [84, 134, 140].

The initial view that the rapid clearance of apoptotic cells *in vivo* does not elicit inflammatory or immune responses in steady-state conditions was expanded by Voll and collaborators [141] who first described that apoptotic cells exert an active and potent immunosuppressive effect on monocytes, promoting the secretion of IL-10 and reducing the release of the proinflammatory cytokines tumor necrosis factor (TNF)-*α*, IL-1*β*, and IL-12. This profound downregulatory effect of apoptotic cells on immunity occurs in professional and nonprofessional phagocytes and in nonphagocytic cells [142].

Several reports have shown that interaction and/or internalization of apoptotic cells by immature DCs does not induce expression of the DC maturation-markers MHC class II, CD40, CD80, CD86, and CD83 *in vitro* or *in vivo*, even after challenge with LPS, CD40 signaling, TNF-*α*, or monocyte-conditioned medium [143–146]. Additionally, DCs that internalize cells in early apoptosis exhibit a selective decrease in the levels of mRNA and secretion of the proinflammatory cytokines IL-1*α*, IL-1*β*, IL-6, IL-12p70, and TNF-*α*, while secreting normal or increased amounts of immunosuppressive transforming growth factor (TGF)-*β*1 and IL-10, even in the presence of LPS [147–149]. DCs that acquire antigens from apoptotic cells efficiently present apoptotic cell-derived peptides to CD4 T cells and cross-present the internalized antigen to MHC class I-restricted CD8 cytotoxic T cells [150–153]. However, DCs exposed to apoptotic cells show a decrease in their ability to stimulate T cells, a phenomenon that seems to be related to the inhibitory effect of apoptotic cells on the amount of expression of MHC and costimulatory molecules, rather than to a defect in the antigen processing function of the APC [132, 144, 145, 154]. 

A report has shown that intestinal DCs with internalized apoptotic cell fragments (from intestinal epithelial cells) travel to mesenteric lymph nodes [155, 156] and DCs with intracellular fragments (probably derived from apoptotic cells) containing a self-antigen produced by parietal cells have been detected near the gastric epithelium and in T cell areas of the stomach-draining lymph nodes [156]. These *in vivo* observations reinforce the concept that internalization of apoptotic cells by DCs in peripheral tissues followed by transportation and presentation of self-peptides to naïve T cells in secondary lymphoid organs plays a critical role in the maintenance of peripheral T cell tolerance [139]. A similar principle could be exploited to restrain the anti-donor T cell response in the transplantation setting. Apoptotic cells carrying the entire repertoire of donor alloantigens can be generated easily *in vitro* by physical (UV-B irradiation) or chemical (incubation with ceramide) treatment of cells expressing MHC class I and class II molecules [121, 125].

Systemic administration of apoptotic cells that carry donor MHC molecules has been used in experimental animal models to inhibit the antidonor response [121, 147, 157], and apparently cells in early apoptosis have advantages for specific targeting of alloantigen to DCs *in vivo* compared to other systems: (i) early apoptotic cells deliver a potent immunosuppressive signal to DCs [143, 145, 147, 154, 158]; (ii) apoptotic leukocytes are a rich source of MHC molecules; (iii) apoptotic cells are easy to prepare [143]; (iv) i.v. administration of apoptotic cells is relatively safe; (v) once injected i.v., blood-borne apoptotic cells are captured efficiently by splenic DCs [147, 153]; (vi) DCs present apoptotic cell-derived allopeptides to T cells [159] and (vii) there is no requirement for prepreparation of DCs loaded with apoptotic cells *in vitro* [160].

In mice, i.v. administration of early apoptotic donor leukocytes before transplantation significantly prolongs the survival of heart allografts [121]. In this model, it has been demonstrated that splenic DCs quickly take up the i.v. injected apoptotic cells, process apoptotic cell-derived peptides onto MHC molecules and mobilize to T-cell areas of the splenic follicle [147]. On the other hand, De Carvalho Bittencourt and collaborators [157] showed in a murine model that i.v. injection of donor apoptotic splenocytes facilitates bone marrow engraftment independently of the origin of the apoptotic bodies. In a recent study, it was shown that administration of donor apoptotic cells decreased the systemic anti-donor T and B cell response and prolonged cardiac allograft survival in mice. Moreover, CD40-CD154 blockade resulted in indefinite graft survival mediated by the generation of Tregs [161].

A better understanding of the mechanisms involved in the interaction of APCs with apoptotic cells could open up new possibilities for the prevention/treatment of the antidonor response or, alternatively, certain autoimmune disorders.

### 4.5. Regulatory T Cells

As described in Section 2.2, the function of Foxp3^+^ regulatory T cells is to maintain immune tolerance and to prevent inflammatory diseases. It has been demonstrated that a lack of Tregs causes autoimmunity and deregulated T cell activation profiles in mouse models and human diseases. The impaired function or homeostasis of Tregs has been implicated in type 1 diabetes, rheumatoid arthritis, multiple sclerosis, and systemic lupus erythematosus [162]. Given the critical function of Tregs in the maintenance of immune tolerance and the specific immunomodulatory mechanisms that can effectively inhibit the targeted effector cell population, their use has been proposed as a therapy to induce specific immune tolerance and to reduce the use of immunosuppressive drugs. In murine models, many groups have used unmanipulated host nTregs or *in vitro* expanded nTregs in combination with immunosuppressive drugs or immune ablation as a strategy to generate immune tolerance and allograft acceptance [163]. It has been demonstrated that the injection of purified or *ex vivo* cultured CD4^+^CD25^+^Foxp3^+^ nTregs significantly reduces GVHD [164, 165] and, in combination with bone marrow transplantation, inhibit alloreactive CD4^+^ and CD8^+^ T cells and prolong allograft survival [166, 167].

It has been demonstrated that nTregs expressing CD4, CD25 and Foxp3 prevent allograft rejection mediated by CD4^+^Foxp3^−^ activated T cells and cytotoxic CD8^+^ T cells; however, nTregs constitute only 5–10% of peripheral CD4^+^ T cells. For this reason, protocols to obtain Tregs have been a subject of intense research in transplantation immunology. Several reports indicate that Tregs can be obtained using different strategies: they can be directly obtained from the host, they can be obtained from the host and expanded *ex vivo*, they can be induced *in vitro* from naïve T cells under appropriate culturing conditions, or they can be induced by polyclonal activation, antigen-specific activation, or allogeneic activation. 

It has been described that Tregs can be generated by culturing naïve T cells with a mixture of immature DCs, mature DCs and B lymphocytes in the presence of a combination of TGF-*β*, retinoic acid, and IL-2 [168, 169]. In addition, alloantigen-specific Tregs can be generated by stimulation of naïve T cells with allogeneic APC and a combination of TGF-*β*, IL-2, and retinoic acid. These alloantigen specific Tregs present immunosuppressive activity *in vitro*; therefore, they could be used as a specific cellular therapy, and in combination with a regimen of low immunosuppression, they could generate immune tolerance to bone marrow allografts. The utilization of alloantigen-specific Tregs as a conditioning protocol could induce the immune tolerance necessary for subsequent solid organ transplantation [166, 170]. 

Regulatory T cells have been used in the generation of mixed chimerism with reduced conditioning regimens, where the peripheral T-cell repertoire of the recipient is maintained largely intact and Tregs of donor origin are crucial to the active suppression [170, 171]. Although Tregs have potent effects in murine allograft models, current evidence indicates that Tregs are not capable of inducing prolonged skin allograft tolerance in unmanipulated recipients [166, 167, 172]. However, the therapeutic use of Tregs is an interesting approach in the development of minimum conditioning protocols for transplants.

### 4.6. Mesenchymal Stromal/Stem Cells

Other immunomodulatory cells with a high potential in future therapies in transplantation are mesenchymal stromal/stem cells (MSCs). It is well known that bone-marrow-derived MSCs have the capacity to migrate to inflammatory sites and regulate the function of most immune cells through direct contact and/or cytokine secretion [54, 173]. 

Recent reports in animal models and human have addressed the potential role of MSCs in the induction and/or differentiation of different immunosuppressive populations. For instance, it has been shown that murine MSCs can suppress heart graft rejection through the induction of Foxp3^+^ T cells and the inhibition of alloantibody production [174]. In keeping with this report, the groups of Maccario and Mougiakakos have demonstrated that human MSCs favor the differentiation of CD4 regulatory T-cell subsets from peripheral-blood mononuclear cells in mixed lymphocyte cultures and prevent skin [175] and semiallogeneic heart rejection [176]. Finally, a recent report has shed light into some of the possible mechanisms involved in the immunosuppressive properties of MSCs as they demonstrated that porcine MSCs inhibit alloreactive T cells through the induction of PGE_2_ and IDO [177]. Thus, although additional efforts are needed to further understand the mechanisms of the observed immunomodulatory properties of MSCs, this population constitutes a promising weapon for future transplant therapies.

## 5. Concluding Remarks

Medical and scientific advances achieved since the first steps of organ transplantation have made it an acceptable resource for human medical care. Nonetheless, since the massive development of organ transplantation near the end of the 20th century, there have been few steps made toward the improvement of allograft survival and pharmacological immunosuppression. As an alternative, cell-based therapy offers the opportunity to induce immune tolerance without the adverse effects associated to pharmacological immunosuppression. Here we have described mechanisms related to allograft tolerance and cellular treatments that have been well characterized for their ability to induce immune tolerance. Dendritic cells, regulatory macrophages, apoptotic cells, regulatory T cells, and mesenchymal stromal/stem cells offer a viable alternative for future use in clinical procedures that could greatly benefit patient survival and quality of life in transplanted patients. The next steps of transplantation immunology will most certainly involve the clinical standardization of dosage, administration, and effectiveness, among other parameters, for the potential therapies discussed here. Efforts are now focused on overcoming the challenges that currently limit the use of cell therapy, either alone or in combination with pharmacological tools, with the goal of breaking through the main causes of failure in the current protocols to achieve organ acceptance.
